# A Rare Genetic Association of an *NPHP2* Mutation With Nephronophthisis in a Child With Bombay Blood Group

**DOI:** 10.14740/jmc5309

**Published:** 2026-06-03

**Authors:** Bassil Leghrouz, Tariq Hindi, Ihab Hilo, Alex Ballout, Musa Hindiyeh

**Affiliations:** a Department of Pediatrics, Augusta Victoria Hospital, Jerusalem, Palestine

**Keywords:** *NPHP2*, Nephronophthisis, Bombay blood group, Pediatric nephrology, Rare disease

## Abstract

Nephronophthisis is a rare genetic disorder affecting the kidneys and is one of the leading causes of end-stage kidney disease in children. The Bombay blood group is an extremely rare blood type that poses serious clinical challenges, particularly when managing patients with chronic kidney diseases. This report described a 3-year-old female patient who presented with severe anemia and kidney impairment, which progressed to end-stage kidney disease. During blood grouping, she was unexpectedly identified as having the Bombay phenotype, complicating her anemia management. Genetic testing revealed two novel pathogenic variants: one in the *NPHP2* gene, associated with nephronophthisis, and another in the *FUT1* gene, which is responsible for the Bombay blood group. The child received treatment involving fluid restriction and diuretic infusions. However, she subsequently progressed to end-stage kidney disease with significant fluid overload and required peritoneal dialysis. Anemia management was achieved through intravenous iron and erythropoiesis-stimulating agents (erythropoietin), eliminating the need for blood transfusions. This case underscores the rare coexistence of nephronophthisis and the Bombay blood group alongside novel mutations, highlighting the critical need for early genetic diagnosis and intervention. It also emphasizes the complexities of managing patients with such rare blood types, which complicate potential transfusion options.

## Introduction

Nephronophthisis (NPHP) is a genetic kidney disorder that leads to progressive kidney disease. It is usually inherited in an autosomal recessive manner and is often seen in families with consanguineous marriages. There are several subtypes, each linked to specific genetic mutations. More than 25 genes, most of which code for proteins needed for primary cilia in kidney cells, are involved in NPHP. The main problem is tubular dysfunction, which causes symptoms like polyuria and polydipsia. It can also lead to severe anemia, poor growth, tubulointerstitial nephritis, cystic kidney disease, and eventually end-stage kidney disease (ESKD), requiring renal replacement therapy [1].

The Bombay blood group (Oh), first identified in India, is an extremely rare blood type characterized by the absence of the H antigen. Individuals with this blood group possess anti-H antibodies, rendering them unable to receive blood from any other blood group (A, B, AB, or O), which poses significant transfusion challenges.

The aim of this report is to describe the coexistence of NPHP, and the Bombay phenotype, and to highlight the clinical implications for transfusion planning and long-term management.

## Case Report

A 3-year-old girl presented to the pediatric emergency department with fever, vomiting, and diarrhea. She was pale, and her complete blood count (CBC) showed a hemoglobin level of 6.6 g/dL. There was no history of hemoptysis, melena, or gross hematuria. The patient was admitted to the hospital for a workup of anemia. The repeated CBC showed a hemoglobin level of 5.8 g/dL, a white blood cell count of 9.8 × 10^3^/mm^3^, and a platelet count of 301 × 10^3^/µL. Blood work on admission is shown in Table 1.

**Table 1:** Blood Work on Admission

Complete blood count (CBC)
WBC: 9.8 × 10^3^/mm^3^ (5–19 × 10^3^/mm^3^)
Hb: 5.8 g/dL (11–13.5 g/dL)
Platelet count: 301,000/µL (150,000–450,000/µL)
Liver function/coagulation
ALT: 86 U/L (30–45 U/L)
AST: 69 U/L (20–60 U/L)
PT: 12 s (11–13.5 s)
INR: 1.0 (0.9–1.3)
PTT: 26 s (25–30 s)
Renal function
Serum creatinine: 2.5 → 2.9 mg/dL (0.3–0.5 mg/dL)
BUN: 37 → 43 mg/dL (5–18 mg/dL)
Vitamin B12: 700 pg/mL (160–950 pg/mL)
Folic acid: 7.5 ng/mL (5–21 ng/mL)
Vitamin D: 11 nmol/L (50–100 nmol/L)
Albumin: 3.8 g/dL (3.4–5.2 g/dL)
LDH: 244 U/L (160–370 U/L)
ESR: 70 mm/h (3–13 mm/h)
TSH: 2.3 µIU/mL (0.5–5.97 µIU/mL)
Free T4: 1.39 ng/dL (0.8–2.8 ng/dL)
PTH: 334 pg/mL (15–32 pg/mL)
Serum electrolytes
Na: 134 mmol/L (135–145 mmol/L)
Cl: 118 mmol/L (90–110 mmol/L)
K: 6.3 mmol/L (3.5–5 mmol/L)
PO_4_: 5.5 mg/dL (4–7 mg/dL)
Ca: 8.6 mg/dL (8.8–10.8 mg/dL)
Mg: 2.5 mg/dL (1.6–2.6 mg/dL)
ABG
pH: 7.29
pCO_2_: 23 mm Hg
HCO_3_^–^: 14 mmol/L
Iron: 35 µg/dL (50–120 µg/dL)
Ferritin: 71 ng/mL (7–140 ng/mL)
TIBC: 350 µg/dL (235–451 µg/dL)
Transferrin saturation: 2.5% (22–39%)
Indirect Coombs test: positive
Direct Coombs test: negative

WBC: white blood cell count; Hb: hemoglobin; ALT: alanine aminotransferase; AST: aspartate aminotransferase; PT: prothrombin time; INR: international normalized ratio; PTT: partial thromboplastin time; BUN: blood urea nitrogen; LDH: lactate dehydrogenase; ESR: erythrocyte sedimentation rate; TSH: thyroid-stimulating hormone; T4: thyroxine; PTH: parathyroid hormone; Na: sodium; Cl: chloride; K: potassium; PO_4_: phosphate; Ca: calcium; Mg: magnesium; ABG: arterial blood gas; pCO_2_: partial pressure of carbon dioxide; HCO_3_^−^: bicarbonate; TIBC: total iron-binding capacity.

When the patient was admitted, she had low urine output, generalized edema, and high blood pressure. Clinical and laboratory results showed signs of chronic kidney disease (CKD). Her kidney function was impaired, and she had metabolic acidosis, hyperkalemia, high parathyroid hormone, and severe anemia, even though her iron levels were normal. An ultrasound showed that both kidneys were small, with the right kidney measuring 5.6 cm and the left 6.5 cm in bipolar length. Both kidneys appeared echogenic with loss of corticomedullary differentiation.

To treat her high potassium and acidosis, she received intravenous calcium gluconate, oral Kayexalate, and intravenous sodium bicarbonate.

The patient’s parents are first cousins. They are both healthy, and there is no family history of kidney or hematological diseases.

NPHP was considered likely because it is common in the area, and the parents are related. A whole-exome sequencing (WES) test was ordered.

As part of anemia management, blood grouping identified the Bombay blood group. Due to the absence of a Bombay blood type donor, anemia was managed with an intravenous erythropoiesis-stimulating agent (ESA) and intravenous iron. The patient’s serum hemoglobin gradually improved, reaching 15 mg/dL after 3 months of treatment. During her illness course, her kidney function test continued to deteriorate, and the urine output became very minimal with significant fluid overload despite the use of diuretics. We decided to insert a peritoneal dialysis catheter and initiate dialysis, which successfully managed her fluid overload. She was discharged on home intermittent peritoneal dialysis (IPD) with regular follow-up.

### Genetic testing result

WES revealed a homozygous pathogenic variant of the *INVS* (*NPHP2*) gene. A genetic diagnosis of autosomal recessive NPHP type 2 was confirmed (the mutated gene: *INVS* (*NPHP2*); variant: NM_014425.3:c.2029del, amino acid change: p.(Ser677ProfsTer66)).

The *INVS* variant c.2029del p.(Ser677ProfsTer66) is a frameshift variant located in exon 13 (of 17) that is predicted to create a premature stop codon and, therefore, produce a truncated protein. To the best of our knowledge, this is a novel variant that has not been previously reported in the literature. It was classified as pathogenic based on the CENTOGENE implementation of the ACMG/AMP/ClinGen SVI guidelines. Pathogenic variants of the *INVS* gene cause autosomal recessive NPHP type 2. (OMIM®: 602088).

WES also showed a novel homozygous missense variant in *FUT1* 35C>A; p.Ala12Asp, reported as a variant of uncertain significance (VUS). This mutation was further confirmed by Sanger sequencing. Sanger sequencing testing revealed that the father, mother, and the proband’s sister were all heterozygous (C/A) carriers of the same *FUT1* mutation. On the paternal side, the grandmother was wild type, whereas the grandfather was a heterozygous carrier. On the maternal side, the grandmother was wild type, whereas the grandfather refused genetic testing. This inheritance pattern across three generations supports autosomal recessive transmission, consistent with the proband’s homozygous genotype and confirmed Bombay phenotype.

## Discussion

This case report describes a 3-year-old girl with chronic kidney disease and severe anemia. Upon investigation, she was found to have the rare Bombay blood group caused by a mutation in the *FUT1* gene, alongside another mutation in the *NPHP2* gene, which is linked to a condition known as infantile NPHP.

This complex combination creates significant challenges for her treatment, particularly as she may need blood transfusions, with her unique blood type requiring careful management to avoid serious reactions. This case underscores the intricate nature of pediatric nephrology, especially in regions where genetic factors, often arising from consanguinity, lead to the prevalence of rare blood types and hereditary kidney diseases. Understanding the genetic factors involved not only clarifies the girl’s clinical situation but also highlights the critical role of genetic testing in diagnosing familial kidney disorders.

NPHP is a common genetic cause of ESKD in children, with its incidence varying widely across populations; for example, the estimated incidence varies from 1:50,000 live births in Finland to 1:1,000,000 in the United States [2]. This genetically heterogeneous disorder is associated with mutations in more than 25 identified genes [3, 4]. In approximately 10–20% of cases, patients exhibit additional features including retinal defects, liver fibrosis, skeletal abnormalities, and neurodevelopmental disorders [1].

Mutations in the *NPHP1* gene are the most common, reported in approximately 20% of cases [5]. Clinically, three clinical subtypes of NPHP have been recognized based on the median age of onset of ESKD: infantile, juvenile, and adolescent/adult [6]. Infantile NPHP or NPHP type 2 (mutation in the *NPHP2* gene) is one of the most significant. It can present in utero with an oligohydramnios sequence (limb contractures, pulmonary hypoplasia, and facial dysmorphisms) or postnatally with severe hypertension and kidney manifestations that progress to ESKD before the age of 3 years. It is characterized by rapid disease progression. Kidney ultrasound findings show moderately enlarged cystic kidneys with cortical hyperechogenicity [7].

The diagnosis of NPHP is suggested by clinical features and confirmed by a positive genetic test. A single-gene mutation can be detected in up to 60% of cases, depending on the cohort composition. However, the absence of a mutation is insufficient to exclude the diagnosis of NPHP. Patients with NPHP typically have a “bland” urinalysis, without evidence of proteinuria, hematuria, or cellular elements, until the late stage, when proteinuria may develop and lead to secondary glomerulosclerosis. To date, treatment options for NPHP remain limited. Blood pressure control is a priority. Management of complications arising from progressive kidney disease, such as anemia, uremia symptoms, and fluid overload, is important alongside preparation for future kidney replacement therapy.

This disease does not recur in a transplant, and kidney transplantation remains the ideal mode of kidney replacement therapy. Managing ESKD in a patient with the Bombay blood group is challenging, especially if the underlying cause of ESKD is NPHP, as these patients are commonly anemic and occasionally require blood transfusions; having the Bombay blood group prevents them from receiving blood from other blood groups. In this case, the patient responded favorably to conservative hematologic management with erythropoietin injections, thereby eliminating the risks of blood transfusion. However, comprehensive long-term planning, including the establishment of a registry for rare blood donors, remains an essential component of care for individuals with rare blood types. One option to help overcome this dilemma is to improve the patient’s hemoglobin to a good level, where his own blood can be stored for any further needs. This involves cooling blood products to very low temperatures (deep-freeze), typically between –80 °C and –96 °C. This process uses cryoprotectants that prevent ice crystal formation, which can damage cells and proteins. Deeply frozen blood products can be stored for several years. Kidney transplantation is another challenge for patients with the Bombay blood group. This difficulty arises because receiving a kidney from a donor with a different blood group carries a high risk of hemolysis and acute kidney rejection. For instance, there is a reported case from India of a patient with the Bombay blood group who successfully received a kidney from a donor with a different blood group after careful, complex strategies. These include exceptionally rare and complex meticulous planning and a precise desensitization protocol with rituximab, plasma exchange, and intravenous immunoglobulin prior to transplantation to prevent hemolytic anemia and rejection of the transplanted kidney [8].

### Conclusions

This case report is the first documentation of a dual diagnosis of NPHP and Bombay blood group. In addition, the two mutations reported in this case are novel. This case highlights the need for specialized clinical management that accommodates genetic considerations and the unique immunological profile of the patient, as the child responded favorably to intravenous iron and ESA. This report aims to discuss the therapeutic implications and interdisciplinary approach to managing such complex cases.

## Data Availability

The data supporting the findings of this study are included within the article. Additional details are available from the corresponding author upon reasonable request, in accordance with patient confidentiality and ethical restrictions.

## Abbreviations

NPHP: nephronophthisis
ESKD: end-stage kidney disease
CKD: chronic kidney disease
WES: whole-exome sequencing
ESA: erythropoiesis-stimulating agent
IPD: intermittent peritoneal dialysis
VUS: variant of uncertain significance
